# P-910. Systemic Antibiotic Use Among the Last 30 Days of Life in Patients with Metastatic Cancer

**DOI:** 10.1093/ofid/ofaf695.1116

**Published:** 2026-01-11

**Authors:** Susannah F Colt, Gavin Hui, Mohana Roy, David R Ha, Jessica Ferguson, Daisuke Furukawa

**Affiliations:** Stanford, Stanford, California; Atropos Health, New York, New York; Stanford, Stanford, California; Stanford Health Care, Stanford, CA; Stanford University, Palo alto, California; Stanford University, Palo alto, California

## Abstract

**Background:**

Many terminally ill patients receive systemic antimicrobials in their last days or months of life, and these rates may be especially high among patients with metastatic cancer. However, the role of antimicrobials at the end of life is limited, and this overuse of antimicrobials represent an important stewardship opportunity. We sought to characterize the antibiotic burden in patients with metastatic cancer to better inform efforts to improve end of life antibiotics use.

**Methods:**

We examined adults with metastatic cancer diagnoses between 2015 and 2024 who received chemotherapy within 30 days of death at a single academic institution, identifying those who also received systemic antibiotics in this time period. Results were stratified by location of primary cancer using ICD 9 and 10 codes, and the National Healthcare Safety Network Antibiotic Use and Resistance module antibiotic groups were used to categorize antibiotics.

**Results:**

Our cohort included 3174 patients, of which 1849 (58.3%) received systemic antibiotics within 30 days of death, with highest utilization seen in patients with hematologic cancer (70.5%) and sarcomas (61.2%). Across cancer types, IV antibiotic use (range 36.2%-56.8%) was higher compared to oral antibiotics (range 32.6%-53.6%) except for head and neck cancers (Table 1). Inpatient antibiotic use was highest in patients with hematologic cancer (53.0%) followed by sarcoma (44.9%) while outpatient antibiotic use was highest in hematologic cancer (34.4%) followed by head and neck (26.8%). Of the inpatient antibiotics, across cancer types, antibiotics for hospital-onset infection (range 17.89%-38.8%) and antibiotics with high *Clostrioides difficile* risk (range 15.38%-39.89%) were the most commonly utilized.

**Conclusion:**

Our results demonstrate high antibiotic utilization in patients with metastatic malignancy during the end-of-life period, with patterns varying based on location of primary cancer. Utilization was especially high in the inpatient setting with high use of broad-spectrum antibiotics. Advance care planning regarding the treatment and prevention of infections, in which thoughtful communication by physicians can help drive alignment of care with patient values, may help limit antibiotic overuse.

**Disclosures:**

Mohana Roy, MD, BMS: Advisor/Consultant

